# HIV healthcare transition outcomes among youth in North America and Europe: a review

**DOI:** 10.7448/IAS.20.4.21490

**Published:** 2017-05-16

**Authors:** Vicki Tepper, Stefanie Zaner, Patrick Ryscavage

**Affiliations:** ^a^ Department of Pediatrics, University of Maryland School of Medicine, Baltimore, MD, USA; ^b^ University of Maryland School of Medicine, Baltimore, MD, USA; ^c^ Department of Medicine, University of Maryland School of Medicine, Baltimore, MD, USA

**Keywords:** HIV, AIDS, healthcare transition, adolescent, youth

## Abstract

**Introduction**: The transition from paediatric to adult care poses risks to the health of young adults living with HIV if unsuccessful, including interruptions in care and poor health outcomes. Evolving best practices in HIV healthcare transition should ideally be informed by real-world qualitative and quantitative clinical healthcare transition outcomes. There has been a recent proliferation of HIV healthcare transition outcome research, largely from Europe and North America.

**Methods**: A literature search was undertaken using the online databases PubMed, Web of Science, and Google Scholar. Medical subject and text word searches were combined for terms relating to HIV, paediatric transition outcomes, and internal and external factors were used to identify peer-reviewed articles.

**Results**: In this paper, we review data on HIV healthcare transition outcomes in North America and Europe. Internal and external factors which may impact the success of HIV healthcare transition are examined. We describe ongoing research efforts to capture transition outcomes in the North America and Europe. Clinical, operational, and implementation science research gaps that exist to date are highlighted. Efforts to improve HIV healthcare transition research through country-level surveillance networks and large multicentre cohorts, including data integration and linkage between paediatric and adult cohorts are discussed.

**Conclusions**: We identified the need for a comprehensive approach to implementing empirically supported protocols to support healthcare transition for ALHIV. While there is limited prospective longitudinal cohort data available at this time, cohorts linking the paediatric and adolescent with ongoing surveillance into adulthood are being developed. Through a review of existing qualitative and quantitative healthcare transition outcomes studies, we identify emerging areas of consensus surrounding healthcare transition research implementation. Successful healthcare transition programmes in Europe and North America often share several characteristics, including implementation of a youth friendly multidisciplinary approach, consistent communication and integration between paediatric and adult care teams, and an individualized approach which is attuned the adolescent’s transition readiness. Moving forward, the voices of youth and young adults living with HIV should be included in the development and evaluation of healthcare transition protocols to ensure that the definition of successful transition reflects all of the stakeholders in the transition process.

## Introduction

Early in the HIV epidemic, mother-to-child HIV transmission (MTCT) contributed significantly to the numbers of children living with HIV in Europe and the United States. Prevention of mother-to-child HIV transmission (PMTCT) and expanded access to combination antiretroviral therapy (cART) in the 1990s led to sharp declines in MTCT in these countries, and current rates of MTCT in these regions are <1% [1]. Now, three decades later, the children of the early HIV epidemic are aging to adolescence and adulthood, yielding a population of young adults entering adult care with a legacy of complex chronic illness and trauma. In addition, a greater proportion of new behavioural HIV infections in the US and Europe are now occurring in youth aged 13–24 (approximately 20% of new HIV infections in the US) [2]. The confluence of these two populations is creating a challenge to HIV providers and healthcare systems as these adolescents begin to transition from paediatric to adult HIV care.

Adolescence is a time of great change and challenge, bridging the chasm between childhood and adult life. For adolescents with living with HIV (ALHIV), this period may be fraught with even greater adversity. One of the distinct challenges faced by emerging adults with chronic illnesses is the transition of their medical care from their long-term paediatric specialty provider to care within the adult healthcare system. Unfortunately, despite the complexity of the healthcare transition process, there is limited evidence in the medical literature to support specific approaches to successful transition. In a recent systematic review of the impact of transition interventions for adolescents with chronic illness, Chu and colleagues summarized the state of the field, *“…few studies have evaluated the impact of transition interventions for adolescents and young adults with chronic illness on the outcome of transfer from pediatric to adult centered care, and… the few studies that have reported on this outcome have been limited by methodological challenges including non-randomization and convenience sampling* [3]*”.*

It is accepted that successful transitions from paediatric to adult healthcare for ALHIV will improve the health outcomes, increase health literacy and self-care skills of emerging adults, which will ultimately result in optimal quality of life. However, the evidence base to support which transition approaches work best is limited by inadequate methodology, challenges with the definition of transition outcomes, a lack of comparison groups, and a paucity of implementation science research. Despite these challenges, a small number of HIV healthcare transition outcomes studies have been published, some of which reveal concerning health consequences to ALHIV as they transition to adult care.

In this paper, we explore the challenges encountered by adolescents and young adults living with HIV, their caregivers and healthcare providers engaging in healthcare transition in Europe and North America. The published outcomes of HIV transition programmes reported in Europe and North America are examined, and we highlight early lessons from these studies and identify evolving clinical, operational, and implementation science research gaps that exist. We discuss efforts to improve HIV healthcare transition research through country-level surveillance networks and large multicentre cohorts, including proposed data integration and linkage between current paediatric and adult cohorts.

## Methods

A literature search was undertaken using the online databases PubMed, Web of Science, and Google Scholar. No restrictions were set on language or publication date; articles were only included if indexed up to 15 January 2017. Medical subject and text word searches were combined for terms relating to HIV, healthcare transition, and paediatric transition outcomes and were used to identify relevant peer-reviewed publications. The abstracts of identified articles were evaluated for relevance. In addition, this procedure was utilized to search for relevant abstracts presented at internationally recognized HIV conferences since 2015.

## Discussion

### Challenges to successful HIV healthcare transition outcomes

*Sociodemographic Characteristics of Transitioning ALHIV in* Europe and North America

In Europe, healthcare transition most often occurs at or near 18 years of age, and adolescent-specific HIV clinics are uncommon [4,5]. However, a recent report describes the introduction of comprehensive United Kingdom (UK)-based HIV transition clinic which utilizes a more individualized approach to timing of healthcare transfer (ages 17–27 years) [6]. The majority of transitioning adolescents in these programmes are living with perinatally acquired HIV. Many of these youth come from families who immigrated from Sub-Saharan Africa. Among the 210 perinatally infected children who had reached adolescence in the French Perinatal Cohort (EPF/ANRS CO10), nearly 40% had mothers of Sub-Saharan African or Caribbean origin [7]. As of 2015, 979 HIV-infected children were in active follow up in the UK Collaborative HIV Paediatric Study (CHIPS) cohort (nearly all with perinatally acquired HIV), 53% were female, 78% were of black African ethnicity, and only half were born in the UK or Ireland [8]. Similar demographic findings were noted in the UK-based Adolescents and Adults Living with Perinatal HIV (AALPHI) cohort (86% Black, 59% born abroad) as well as in two Scandinavian cohorts (where 61–79% of ALHIV were born abroad) [9–11].

In the United States, HIV healthcare transition usually occurs at a somewhat older age; often when youth have reached their mid-twenties. As a result, the transitioning population includes higher relative proportions of behaviourally HIV-infected youth, populations which may differ from those with perinatally-acquired HIV in meaningful ways. Though mostly native-born, a large proportion of ALHIV with perinatally acquired HIV in the US are ethnic minorities living in urban, socio-economically disadvantaged communities. In a US-based prospective, multicentre surveillance study of long-term outcomes in HIV-infected children and adolescents (IMPAACT P1074), among 1173 patients alive as of 2014 (87% of whom acquired HIV perinatally), 58% were African American, 28% were Hispanic, and 11% were White [12]. In addition, youth aged 13–24 years accounted for 22% of HIV diagnoses in 2014 and represented the fastest growing risk group for new HIV infection [2,13]. Among this group, 72% identify as young Black men who have sex with men (BMSM). A recent modelling study reported that half of all Black MSM will become infected with HIV in their lifetime, highlighting the urgent need to engage this population in HIV care and prevention efforts [14].

Healthcare transition challenges across these populations may be broadly conceptualized as comprising internal factors, which are unique to an individual adolescent and/or sub-population, and external factors, or provider and/or structural circumstances over which the adolescent has little or no control. ALHIV likely experience healthcare transition challenges derived from unique combinations of these internal and external factors.

## Internal factors

### Childhood trauma

At the time of healthcare transition, most adolescents and young adults living with HIV have navigated years of life stressors, including serious illness and hospitalization, HIV disclosure to partners and friends, often the loss of one or both parents, and trauma associated with living in limited-resource communities affected by violence. The scope of parental loss in the perinatal HIV population cannot be overstated. Forty-six per cent of ALHIV in the French EPF/ANRS CO10 cohort had a deceased mother, and one-third lived with non-relative caregivers [7]. Thirty-five per cent of patients enrolled in the UK-based AALPHI cohort had a deceased parent [9]. In a US-based cohort of 59 transitioning adolescents with perinatal HIV, 61% had a deceased mother and 51% had a deceased father [15]. An even higher rate of parental death (74%) was noted in a cohort of mostly perinatally infected adolescents in Sweden [10]. The patients may also be thrust into the role of caretakers for sick parents and may be biological parents themselves [15,16]. Childhood trauma is, unfortunately, not limited to the perinatal HIV population. One-third of patients in a cohort of 104 patients with primarily behaviourally acquired HIV in a US-based healthcare transition programme had experienced childhood sexual abuse [17]. Finally, for those youth in immigrant families, healthcare transition may occur while attempting to adjust socially and culturally to their new surroundings [18].

### Caregiver role in healthcare transition

The caregiver’s role in the HIV healthcare transition process is notable among youth living with perinatally acquired HIV. The parents and/or caregivers of adolescents living with perinatally acquired HIV have been closely involved in all aspects of their care since their diagnosis. As a result, these adolescents may not fully understand their healthcare options as someone else had been making the decisions for them, and their voice may have not been solicited or heard. Adolescents living with perinatally acquired HIV may feel passively involved in their healthcare decisions given their caregiver’s historical role in healthcare decisions [19].

### Attachment to paediatric healthcare team

As a result of their longstanding relationship, perinatally infected adolescents and their families may view the paediatric care team as extended members of their family, especially in the context when family members have been lost [20]. Leaving the paediatric practice may be seen as a loss of a significant relationship and support in addition to a change in healthcare system. A study focusing on transitioning HIV-infected youth in a UK-based programme several patients identified attachment to the paediatric team as a barrier to transition, and ongoing communication with the paediatric team often occurred post-transition [21]. The paediatric care team often experiences a similar attachment through decades of shared experiences and struggles [22].

### Adolescent development

Healthcare transition occurs during the dynamic period of adolescent development. The normal developmental process of adolescence involves physical, cognitive, and psychosocial changes during which identity is formed, autonomy is established (including completing education and establishing a vocation), intimate relationships are developed, and abstract thought is incorporated into cognitive reasoning [23,24]. Importantly, traumatic events, as well as acute to chronic physical and mental distress, can halt or alter this normal developmental process and delay the completion of these adolescent developmental tasks [23]. During adolescence, certain developmental characteristics, including concrete reasoning, increased sense on invulnerability, and strong influence from peer networks, may lead to increased risk for disengagement from care into early adulthood [13,25].

### Stigma

HIV is a highly stigmatized illness and many ALHIV report that HIV-associated stigma and disclosure to sexual partners, friends, and family is a barrier to engagement in adult care and quality of life [15,26,27]. HIV-associated stigma is magnified by the reliance on peer-networks and the need to feel “normal” [13,15,19]. Finally, homophobia, racism, and sexual identity-associated stigma are well-established barriers to engagement in care among behaviourally infected transitioning adolescents and young adults [28].

### Neurocognitive disease

Many adolescents and young adults with perinatally acquired HIV experience high rates of neurodevelopmental deficits, particularly among those were born prior to the inception of cART [29]. These deficits appear to correlate with nadir of immune suppression, exposure to early, toxic antiretroviral regimens, and early childhood encephalopathy [30–32]. There is emerging understanding of the effect of childhood CNS HIV disease on learning and communication disorders, as well as executive functioning in adolescence and young adulthood [30,33]. In addition, the adolescent brain continues to develop into early adulthood, affecting learning and memory reinforcement and complex processing. As such, there is a growing awareness of the potential neurocognitive effects of HIV among adolescents with behaviourally acquired HIV [33]. Whether associated with neurocognitive impairment or not, poor health literacy is another barrier to successful transition [34].

### Mental health

Adolescents with both behaviourally and perinatally acquired HIV have higher rates of mental health diagnoses when compared to their HIV-negative peers [19,35], in part reflecting their experiences with childhood trauma (including parental death and drug use), illness, and stigma, as well as other sequelae of longstanding HIV through childhood [36]. Reports of the prevalence of mental health disorders in US-based cohorts of adolescents with perinatal HIV have ranged from 25% to 61% [36,37]. Importantly, the prevalence of mental health disorders among perinatally infected children and adolescents appears in increase as they age to adulthood [38–41]. Prevalence of mental health disorders among transitioning youth with behaviourally acquired disease also appears to be high when compared with the general population [42]. In a small study of transitioned young adults with perinatal HIV, one-third reported a lack of access to emotional support services in adult care [15]. Though mental health disorders among perinatally HIV infected youth have not been studied as a risk factor for unsuccessful transition, both behavioural and mood disorders have been associated with poor cART adherence and lack of virologic control [37].

### Pregnancy

ALHIV face similar sexual health challenges as their non-HIV-infected peers, including pregnancy. In a study examining transition practices in an Adolescent Medicine Trials Network for HIV/AIDS Interventions (ATN) clinic, it was observed that young women with HIV who had been pregnant appeared to have an easier transition to adult medical care. It was suggested that the experience of receiving prenatal care in a different clinic, as well as additional social services offered to the pregnant woman, facilitated the movement from paediatric to adult care for this group of transition aged patients [43].

### Substance use

Given the development of autonomy, limited self-perception of risk, and close dependence on peer groups, adolescence is a time of increased risk for substance use. In addition, HIV-infected adolescents with mental health disorders have a higher risk for substance abuse [36]. Among those with behaviourally acquired HIV, substance use is a well-described risk factor for acquisition of HIV disease and impairs cART adherence and retention in care [44].

### Socioeconomic barriers

Youth living in economically disadvantaged areas are less likely to make appointments and demonstrate consistent adherence to antiretroviral therapy [45]. Poverty can impact prioritizing competing needs such as food and housing over adherence to care, and may also affect logistical needs in the healthcare transition process, including transportation to clinic, and insurance [17,19,46–48]. Of note, unlike most of Europe which have variably implemented universal health coverage, adolescents in the US are at risk for losing insurance when reach 18 years of age. In the US, although the Affordable Care Act has provided access to a wider range of insurance coverage, negotiating the system can be challenging [49]. In a study of perinatally infected young adults’ post-transition, half of participants who had self-discontinued ART cited insurance as the primary reason [15]. Finally, the geographic mobility of adolescents cannot be underestimated, creating a barrier to long-term engagement in a single HIV programme [50].

## External factors

### The paediatric clinic

Paediatric and adolescent HIV care is characterized by an emphasis on multidisciplinary on-site care with a youth friendly environment, a family-centred focus, and psychosocial support which attends to adolescent developmental needs [19]. Many adolescents with HIV (both perinatal and behaviourally acquired) develop strong and longstanding relationships with their care team, often seeing them as members of their family, especially in the context of parental loss [43,51]. As such, paediatric providers may be reluctant to disengage from adolescent and young adults’ care [52].

### The adult clinic

Typically, paediatric and adult HIV clinic environments differ significantly. Adult HIV clinics are often more formal and business like in approach, with limited scheduling flexibility, more patient- and disease-focused care, less co-located specialty care, and fewer youth-friendly services [34]. These characteristics may explain the poor outcomes of ALHIV seen in adult care. In a US-based retrospective study of youth aged 17–24 attending an adult HIV clinic (many of whom had never been in paediatric care), youth had inferior rates of viral suppression and retention in care compared to adults matched on several disease and demographic criteria [40]. Transitioning youth have identified fear of the adult clinic environment as a barrier to healthcare transition and have described difficulties after transfer to adult clinics in dealing with insurance issues and longer wait times [51,53]. Encouragingly, the incorporation of youth-friendly structures of care appears to improve rates of retention in care [24]. Engaging and training adult providers in adolescent and LGBT-friendly HIV care models may be useful as many adult providers lack the expertise or will to provide youth-friendly services in the adult setting [43,54].

### Paediatric and adult provider communication

The degree to which the adult and paediatric healthcare teams communicate and collaborate may affect the success of HIV transition. In a qualitative study establishing HIV healthcare transition best practices, providers cited paediatric/adult communication as an essential element of successful transition [55]. Unfortunately, the experiences of transitioning ALHIV suggests that paediatric and adult provider communication is not universally optimized [15,51]. The need for coordinated communication between paediatric and adult providers has recently been codified in a statement by the American Academy of Paediatrics Committee on Paediatric AIDS [13].

### Heterogeneity of healthcare transition programmes

Though provider-directed guidelines on HIV healthcare transition exist in the US [55], and HIV transition programmes have begun to share protocols [17,56], there remains a lack of universal consensus on HIV transition best practices [24]. Despite the known challenges to HIV transition outlined above, guidelines remain largely driven by expert opinion and there is currently no evidence on what interventions will engender a successful transition. This uncertainty reflects a lack of agreed upon definitions of successful healthcare transition outcomes and lack of research addressing operational and implementation science.

## HIV healthcare transition outcomes

Historically, limitations of HIV transition research have included relatively small samples sizes, varying methodologies, and a heavy reliance on only qualitative research. In addition, most studies examined outcomes exclusively among perinatally infected adolescents and young adults. Studies of outcomes of HIV transition can be categorized into three basic areas: studies examining barriers and expectations prior to transition, descriptive analyses of provider practice during transition, and retrospective qualitative and quantitative descriptions of transition outcomes. As qualitative outcomes associated with expectations pre-transition and provider practices have been published elsewhere [57], we will focus on the literature describing post-transition outcomes.

### Post-healthcare transition qualitative outcomes studies

Wiener et al. interviewed 59 post-transition youth in the US about their healthcare transition experience. Forty-five per cent of the youth who participated in the interviews reported finding the transition more challenging than anticipated [15]. Miles et al. interviewed 7 youth who had completed transition in London [21]. Three of the seven patients experienced difficulty and delay in the transition due to attachment to paediatric providers. In contrast, Bundock et al. found high levels of satisfaction with the transition process in a unique UK-based programme which provided multidisciplinary youth-friendly services with shared paediatric and adult provider care [58]. Kakkar et al. conducted post-transition interviews among 25 patients who had transferred from a Montreal-based paediatric HIV programme to adult HIV care and found that the majority would have preferred deferring transition to a later age (all transferred at 18 years), and many expressed concerns about adapting to the inflexibility of the adult clinic environment [59]. These four studies were largely comprised of perinatally infected adolescents, whereas Valenzuela et al. interviewed ten behaviourally infected young adults (aged 24–28) who also described several frustrations with adapting to the logistics and atmosphere of the adult clinic environment [51].

### Post-healthcare transition quantitative outcomes studies

Though there remains no consensus on what outcomes constitute a successful transition, researchers have identified several potential clinical outcomes to quantitatively measure transition success, including retention in adult care, ART adherence, HIV viral suppression, immunological (CD4) stability, and mortality in adult care. Studies which have examined one or more of these healthcare transition outcomes are described below and outlined in Table 1, where transition outcome categories are more easily compared.

**Table 1.** Clinical Outcomes Following HIV Health Care Transition in the Medical Literature.

Study [Ref] (Region)	Population	Median age at Transfer (years)	Post-HCT Retention	Post-HCT cART Adherence	Post-HCT HIV Suppression	Post-HCT CD4 Change	Post-HCT Mortality
Maturo, 2015 [17] (US)	N=38 BA-HIV	NA	18 (47%) completed transfer to adult care^b^	NA	NA	NA	NA
Fish, 2014 [60] (UK)	N=14, PA-HIV	17	NA	At transfer: 64% taking HAART At death: 36% taking HAART	14% with documented suppression at last evaluation	At transfer: median CD4: 120 cells/uL At death: median CD4: 27 cells/uL	100% (by study design)
Ryscavage, 2016 [42] (US)	N=50 19 PA-HIV 31-BA-HIV	24.5	50%^a^	NA	Pre-transfer: 36% Post-transfer: 57% (p=NS)	Pre-transfer: 347 cells/uL Post-transfer: 351 cells/uL	0%
Hope, 2016 [61] (UK)	N=211 PA-HIV	17.6	88%	NA	Pre-transfer: 43% Post-transfer: 63% (p=<.001)	Pre-transfer: 450 cells/uL Post-transfer: 420 cells/uL *Rate of decline decreased post transfer	4.3%
Righetti, 2015 [62] (Italy)	N=45	8.8	84%^b^	NA	Post-transfer: 73%	NA	NA
Kakkar, 2016 [59] (Canada)	N=45 PA-HIV (25 consented to study)	18	76%^c^	60% reported less than “excellent” adherence	Pre-transfer: 60%	Pre-transfer: CD4>500 cells/uL: 41%Post-transfer: CD4>500 cells/uL: 29%	8.9%
Weijsenfeld, 2016 [11] (Netherlands)	N=59 78% PA-HIV 7% BA-HIV 12% unknown	18.8	86% Mean missed appts increased from 0.2/yr to 0.3/yr after HCT (p<.001)	NA	NA	NA	NA
Westling, 2016 [10] (Sweden)	34 91% PA-HIV	19	NA	Pre-transfer: 88% prescribed HAART	Pre-transfer: 90% Post-transfer: 92%	NA	NA

PA-HIV: perinatally-acquired HIV; BA-HIV: behaviorally acquired HIV; HAART: Highly active antiretroviral therapy; HCT: health care transition ^a^Retention was defined as the completion of at least two visits over 12 months following linkage to adult care. ^b^Definition of post-HCT not defined. ^c^Retention was defined as at least one physician visit within 6 months of the interview.

#### European studies

Fish et al. described the clinical characteristics of fourteen perinatally infected young adults previously enrolled in the CHIPS cohort and who died in adult care after transition in fourteen clinics in the UK [61]. The median CD4 count at time of death was 27 cells/uL, a decline from 120 cells/uL at the time of transfer from paediatric care. Only 36% were taking cART prior to death, and 14% had achieved HIV viral suppression. Sixty-four per cent also carried a mental health diagnosis [60]. This study underscored the significant clinical and psychosocial challenges associated with caring for perinatal ALHIV in adult care settings.

Hope, et al. examined post-transition outcomes among 211 adolescents with perinatally acquired HIV in the UK-based CHIPS programme, 57% of who transitioned within the same hospital, and 55% of who had a staged transition between one or more clinics. They found that those who changed hospitals for adult transfer had higher odds of clinic non-attendance or death [61]. Encouragingly, rates of viral suppression and CD4 decline improved after transfer in this cohort.

Righetti et al. described survey-based post-transition outcomes among 45 HIV+ adolescents attending a multidisciplinary programme which was transitioned to an adult programme in Genoa, Italy [62]. Due to the nature of this programme model, many patients transitioned as children (median age 8.8 years). Though difficult to extrapolate given these unique circumstances, 84% successfully were retained in the adult programme.

Weijsenfeld et al. captured post-transition outcomes in a cohort of 59 Dutch adolescents with predominantly behaviourally acquired HIV [11]. Like the UK and much of Europe, HIV transition typically occurs at 18 years in the Netherlands and paediatric HIV occurs at four paediatric HIV specialty clinics throughout the country. These clinics have adopted transition protocols which emphasize transition readiness assessment and training, as well as pre-transition involvement of the adult provider [11]. Though eighty-six per cent of patients in this cohort were retained in care, rates of missed appointments increased significantly from pre- to post-transition. Virologic failure was significantly higher in the period shortly following transfer to adult care and was significantly associated with lower educational attainment and lower medication adherence autonomy at transfer. Importantly, those with durable viral suppression in paediatric care were unlikely to develop viral rebound in adult care [11]. These results highlight the importance of identifying and addressing pre-transfer adherence risks to improve outcomes post-transition.

Sainz et al. performed a cross-sectional study examining pre- and post-transition immunologic and virologic outcomes among the Spanish Cohort of the Spanish Paediatric HIV Network (CoRISpe) [63,64]. Variables were recorded at last paediatric visit, one year post-transition, and at the end of the follow-up period (December, 2013). Among 147 perinatally infected patients with available longitudinal data, rates of viral suppression improved from 55% pre-transition to 79% one year post-transition, though overall improvements in viral suppression may have reflected general improvements in cART access and efficacy over the long period of the study (1998–2013). Loss to follow up and mortality was subsequently noted to be 13.9% and 2%, respectively [63,64].

Westling et al. performed a pre-transition cross-sectional study and 2-year follow up among 34 HIV+ adolescents (91% perinatally-infected) who underwent transfer to adult care following the development of the Treatment Outpatient Clinic, a co-located paediatric and adult multidisciplinary clinic in Sweden, after which youth may stay in adult care in the same physical space [10]. In this optimized setting, pre- and post-transition viral suppression was high (90%, 92%, respectively). Of note, adult care retention was not assessed in this study design.

#### North American studies

Maturo et al. provided the first description of post-transition outcomes in a US-based cohort of 38 patients with behaviourally acquired HIV attending a youth-friendly adolescent clinic with a formal healthcare transition protocol [17]. This 5-phase protocol (“Moving” Out) involves multidisciplinary participation by the care team and begins with early discussion regarding transition followed by adult HIV provider care within the adolescent clinic, followed by transfer to adult HIV clinic after readiness assessment is completed. At the time of study assessment, 18 of the 38 patients had completed the 5 phases and had moved on to adult care. Of the remainder, three had not yet completed the five phases, nine had relocated out of the area, four wilfully disengaged from care, and 3 were lost to follow up. Among the latter two groups who did not successfully transition, no association was found with substance abuse, mental health disease, cART adherence, and pregnancy, though the sample size was likely too small to detect such an association [17].

Ryscavage et al. examined clinic retention, ART use, viral suppression, and CD4 trends following transition among 50 (19 perinatal, 31 behavioural) HIV-infected young adults [42]. Though 86% of patients were successfully linked to adult care in this study, only 50% were retained in adult care at 12 months. Pre- and post-transition HIV suppression rates and CD4 counts did not differ and there was no difference in outcomes between those with perinatal and behaviourally acquired HIV, though the study number was small [42]. Of note, mental health diagnoses were high in both perinatal and behaviourally infected groups (53% vs. 55%) and though there were numerically higher rates of substance abuse in behaviourally vs. perinatally acquired HIV groups (32% vs. 58%) this did not reach statistical significance [42].

Kakkar et al. interviewed 45 patients who had transferred from a Montreal-based paediatric HIV programme to adult HIV care. These interviews were conducted a median of 3.6 years post-transfer. Of these 45 patients, 25 consented to the study, 8 were lost to follow up, 8 declined participation, and 4 had died (mortality of 8.8%) [59]. Seventy-six per cent of patients who consented to interview were still retained in adult care, though this number does not reflect the additional 16 patients of the original 45 who were lost to follow up and/or declined participation. Ninety-per cent of those with undetectable viral loads pre-transfer had undetectable viral loads at 1 year post transfer [59].

### Early lessons from HIV healthcare transition outcomes studies

Despite the relatively small sample sizes, variable methodology, disparate transition success measures, retrospective designs, lack of representation from behaviourally infected adolescents, and limited external validity due to variations in transition practice, the emerging literature on HIV healthcare transition has focused on several outcomes. The two most commonly utilized transition outcomes have been retention in adult care (eight of nine studies cited above) and HIV suppression (eight of nine studies cited above). CD4 + T cell changes and cART adherence have been less frequently examined, and the results of those studies which did include these outcomes suggest that they may be less useful instruments for gauging transition success. Mortality, though rare, appears to be an important transition variable, specifically as a means to examine predictors of the most catastrophic healthcare transition outcomes. Additionally, pre-transition outcomes, including retention in paediatric care and viral suppression, may be markers of the likelihood of post-transition success [11,63,64]. Finally, the patient and care team experience remain essential to healthcare transition research, allowing contextualization and closer examination of the transition experience.

In addition to transition categorical outcomes, choices, there are several meaningful lessons from the emerging HIV healthcare transition literature. First, the transitioning ALHIV population is complex and heterogeneous, and the possible consequences of poor transition include lack of viral suppression, immune deterioration, and death [61,60]. Second, transition programmes which have reported favourable results share several common characteristics; a multidisciplinary approach, early and integrated involvement of an adult care team, optimal communication between paediatric and adult care teams, and an individualized approach which is attuned the adolescent’s transition readiness. Third, results from qualitative studies, as well as those from successful transition programs, highlight the importance of implementation of youth-friendly services as part of the post-transition care structure [34]. Finally, because of the complexity of needs among this population, transition of care should include concurrent transition of mental health services, substance abuse treatment, and case management delivered by multidisciplinary teams. Many of these themes have been corroborated by qualitative research of HIV healthcare providers and expert panels [43,65,66].

## Unaddressed gaps in HIV healthcare transition research

Despite the growth of the literature on HCT outcomes, there is an urgent need to study transition outcomes and transition models for behaviourally infected adolescents, particularly as there is incompletely explored evidence that behaviourally infected adolescents may be less likely to be retained in adult care either alone or compared to those with perinatally acquired HIV [40,67]. In addition, there is evidence that structural barriers to accessibility and availability of youth-centred care may significantly impact the degree to which adolescents remain engaged in HIV care [68]. To that end, there remains a critical need to address several operational and implementation science research gaps as outlined in Table 2. Finally, there is a need for structural realignment of large paediatric and adult HIV cohorts to include unified data collection methodology and data integration. These specific challenges are listed below.

**Table 2.** Research gaps in operational/implementation science.

Effective strategies supporting adherence among transitioning adolescence
Interventions to promote retention in care during and after HCT
Programmatic HCT needs of perinatal vs. behaviorally-infected adolescents
Measurement of ART status before, during, and after the HCT process
Optimal age for HCT
Evidence assessing effective programs which support emotional, mental and social outcomes for adolescents and young adults
Evidence supporting predictors of a successful transition (including adolescent-perceived outcomes)
Examination of strategies to facilitate adult clinician buy-in in the HCT process
Assessment of critical youth-friendly services for transitioning adolescents
Long term clinical and psychosocial outcomes following HCT

Adapted from the Collaborative Initiative for Paediatric HIV Education and Research Adolescent HIV Transition Workshop, 2015. [48] Abbreviations: HCT: health care transition

### Need for improved surveillance and data harmonization across cohorts and surveillance systems

There is a recognized need for improved surveillance of paediatric and adult cohorts through the transition process. Linkage of these cohorts will allow for long term, seamless monitoring of clinical and transition outcomes in order to inform HIV clinical and policy-level decisions in the future. Among paediatric and adolescent cohorts, researchers have identified the need for HIV disclosure in the consent process as a potential barrier to enrolment of adolescent participants [50]. In the US, economically disadvantaged communities, especially African Americans, may also harbour a mistrust of biomedical research which may deter them from enrolment in longitudinal cohorts [69]. Unfortunately, many country-level surveillance networks as well as research-based paediatric and adult cohorts are not integrated, and data collection throughout these networks may be disaggregated. Recent efforts are under way in the Europe and the US to harmonize the collection of transition-associated outcomes, and are discussed below.

### European multicentre cohorts

A recent survey study of HIV cohort studies participating in the EuroCoord Network of Excellence was performed in order to ascertain the number and distribution of patients with perinatally acquired HIV, as well as to examine the degree of seamless linkage between individual European paediatric and adult HIV cohorts [70]. This study identified 8,229 patients with perinatally acquired HIV in 16 European countries. Only four countries (Denmark, Greece, Netherlands, and Romania) collected paediatric and adult cohort data in the same database [70]. Several other European countries, however, have begun to make progress in the linkage of paediatric and adult HIV cohort data, and are discussed below.

The UK has had a comprehensive observational active surveillance system for paediatric HIV for the last 25 years through the National Study of HIV in Pregnancy and Childhood (NSHPC) [50]. In addition, two large longitudinal adolescent HIV research cohorts have been developed. The Adolescents and Adults Living with Perinatal HIV (AALPHI) cohort is a longitudinal study of HIV-infected (N = 300) or affected (N = 100) adolescents, aged 13–21, which is examining 5 key domains: neurocognitive, cardiac, metabolic, sexual and reproductive health and anthropometry and bone composition [9]. This study will be able to provide general surveillance data to examine retention outcomes in the transition process. The Collaborative HIV Paediatric Study (CHIPS) is a multicentre cohort study of HIV-infected children in the UK and Ireland, established in 2000 [9]. CHIPS has developed an effective surveillance system through collaboration with the NSHPC, capturing nearly all HIV infected children (N = 1934 as of June 2015) receiving care in the UK and Ireland from 2006 [9]. CHIPS is currently re-consenting participants aged ≥16 years into the CHIPS+ cohort for long-term follow up [9]. Many individual European countries also have operational longitudinal research paediatric cohorts such as the French ANRS Perinatal Cohort (EPF) and the Spanish Cohort of the Spanish Paediatric HIV Network (CoRISpe) [7,71]. These and other European paediatric cohorts are in various states of integration with longitudinal adult HIV cohorts. The Swiss HIV Cohort is an example of a fully integrated country-wide cohort incorporating harmonized paediatric and adult HIV data collection [72]. In addition, the European Pregnancy and Paediatric HIV Cohort Collaboration (EPPICC), composed of 25 cohorts in 15 countries, is overseeing the integration of paediatric and adult HIV surveillance in several countries, some of which (such as Spain, Belgium, and Switzerland) have already begun to establish strong surveillance data integration [50]. These networks are encountering challenges, including the need for re-consent of adolescents into adult cohorts, and the use of differing pseudo-anonymized identifiers in different cohorts [50].

### US multicentre cohorts

Prospective longitudinal cohorts of older adolescents transitioning into adulthood are now being followed in the US. The Adolescent Medicine Trials Network for HIV/AIDS Interventions (ATN) has developed the CATCH (Comprehensive Assessment of Transition and Coordination for HIV-Positive Youth as They Move From Adolescent to Adult Care) cohort (ATN 135), which will capture medical and appointment records data across the transition spectrum, and prospectively explore pre- and post-transition experiences of ALHIV (age 18–24 years) [73]. In addition to clinic retention data, investigators are collecting pre- and post-transition HIV viral loads and measures of physical and psychosocial health status [70]. The Pediatric HIV/AIDS Cohort Study (PHACS) Adolescent Master Protocol (AMP Up) has enrolled 451 HIV-infected youth who were 7–16 years of age at enrolment [74]. Nearly 20% of these youth are now 18 years of age and older. These older adolescents are being approached to re-consent for enrolment in AMP-UP, a prospective study to examine the long-term impact of HIV and ART on youth as they age into adulthood [74]. Investigators will be collecting individual, disease-related, and social factors contributing to ART and clinic adherence and healthcare behaviours, including transition success. Unfortunately, neither AMP-UP nor ATN-135 has integrated with existing adult HIV cohorts at this time. Additionally, there are no integrated US national surveillance programs which capture outcomes through the transition process.

## Conclusions

We have identified the need for a comprehensive approach to planning and implementing rigorous empirically supported protocols to support transition for ALHIV. Published studies point to several potential key measures which could serve as markers of successful healthcare transition models including viral load suppression and long-term retention in adult care. While not all are empirically supported, we have identified programmatic approaches which have been shown to improve the transition process for ALHIV. These approaches include: a youth friendly multidisciplinary approach, early and integrated involvement of an adult care team, optimal communication between paediatric and adult care teams, and an individualized approach which is attuned the adolescent’s transition readiness. While there is limited prospective longitudinal cohort data available at this time, cohorts linking the paediatric and adolescent with ongoing surveillance are being developed. These diverse multicentre cohorts of ALHIV will allow programmes to systematically evaluate the efficacy of models of transition that work best for specific populations of ALHIV. The voices of youth and young adults living with HIV should be included in the development and evaluation of transition protocols to ensure that the definition of successful transition reflects all of the stakeholders in the transition process.

## References

[1] Fowler MG, Gable AR, Lampe MA, Etima M, Owor M. Perinatal HIV and its prevention: progress toward an HIV-free generation. Clin Perinatol. 2010;37(4):699–70. 21078445 10.1016/j.clp.2010.09.002

[2] Centers for Disease Control and Prevention. HIV and Youth [cited 2016 9 2]; Available from: http://www.cdc.gov/hiv/group/age/youth/

[3] Chu PY, Maslow GR, Von Isenburg M, Chung RJ Systematic review of the impact of transition interventions for adolescents with chronic illness on transfer from pediatric to adult health care. J Pediatr Nurs. 2015;30:e19–27. 10.1016/j.pedn.2015.05.022 PMC4567416 26209872

[4] Collaborative HIV Paediatric Study (CHIPS) Annual Report 2014/15 [cited 2016 8 29]; Available from: http://www.chipscohort.ac.uk/documents/2015%20CHIPS%20Annual%20Report.pdf

[5] Foster C, Judd A, Tookey P, Tudor-Williams G, Dunn D, Shingadia D, et al Young people in the United Kingdom and Ireland with perinatally acquired HIV: the pediatric legacy for adult services. AIDS Patient Care STDS. 2009;23(3):159–66. 19866533 10.1089/apc.2008.0153

[6] Kim S, Kim SH, McDonald S, Fidler S, Foster C Transition to adult services - a positive step. AIDS Care. 2016 12 15 1–5. [Epub ahead of print] 10.1080/09540121.2016.1268672 27973920

[7] Dollfus C, Le Chenadec J, Faye A, Blanche S, Briand N, Rouzioux C, et al Long-term outcomes in adolescents perinatally infected with HIV-1 and followed up since birth in the French perinatal cohort (EPF/ANRS CO10). Clin Infect Dis J. 2010;51(2):214–24. 10.1086/653674 20536367

[8] Collaborative HIV Paediatric Study (CHIPS). Summary Data to the End of June 2015 [cited 2016 9 2]; Available from: http://www.chipscohort.ac.uk/summary_data.asp#f1

[9] Le Prevost M, Judd A Life after CHIPS: CHIPS and AALPHI update. Paper presented at: 9^th^ Annual Conference of the Children’s HIV Association (CHIVA); 2015 May 22; Leicester.

[10] Westling K, Navér L, Vesterbacka J, Belfrage E Transition of HIV-infected youths from paediatric to adult care, a Swedish single-centre experience. Infect Dis (Lond). 2016;48(6):449–52. 26950534 10.3109/23744235.2016.1143964

[11] Weijsenfeld AM, Smit C, Cohen S, Wit FW, Mutschelknauss M, Van Der Knaap LC, et al Virological and Social Outcomes of HIV-Infected Adolescents and Young Adults in The Netherlands Before and After Transition to Adult Care. Clin Infect Dis. 2016;63(8):1105–12. 27439528 10.1093/cid/ciw487

[12] Mirani G, Williams PL, Chernoff M, Abzug MJ, Levin MJ, Seage GR 3rd, et al Impact: changing trends in complications and mortality rates among US youth and young adults with HIV Infection in the Era of combination antiretroviral therapy. Clin Infect Dis. 2015 12 15;61(12):1850–61. 26270680 10.1093/cid/civ687 PMC4657532

[13] Flynn PM, Aldrovandi GM, Chadwick EG, Chakraborty R, Cooper ER, Schwartzwald H, et al Committee on pediatric Aids. Transitioning HIV-infected youth into adult care. Pediatrics. 2013;132:192–7. 23796739 10.1542/peds.2013-1073

[14] Hess K, Hu X, Lansky A, Mermin J, Hall HI Estimating the lifetime risk of a diagnosis of HIV infection in the United States. Abstract presented at Conference on Retroviruses and Opportunistic Infections (CROI), 2016 2 22-25, Boston, MA.

[15] Wiener LS, Kohrt BA, Battles HB, The PM HIV experience: youth identified barriers for transitioning from pediatric to adult care. J Pediatr Psychol. 2011;36(2):141–54. 20040607 10.1093/jpepsy/jsp129 PMC3042597

[16] Foster C, Fidler S Optimizing antiretroviral therapy in adolescents with perinatally acquired HIV-1 infection. Expert Rev Anti Infect Ther. 2010;8(12):1403–16. 21133665 10.1586/eri.10.129

[17] Maturo D, Powell A, Major-Wilson H, Sanchez K, De Santis JP, Friedman LB, et al Young adults with HIV infection to adult care: pilot testing the “Movin’ Out” transitioning protocol. J Pediatr Nurs. 2015;30(5):e29–35. 10.1016/j.pedn.2015.06.013 26276460

[18] Foster C CHIVA Guidance on Transition for adolescents living with HIV. [cited 2016 8 21]; Available from: www.chiva.org.uk/files/1214/2857/8197/transition.pdf

[19] Dowshen N, D’Angelo L Health care transition for youth living with HIV/AIDS. Pediatrics. 2011;128(4):762–71. 21930548 10.1542/peds.2011-0068

[20] Jacob S, Jearld S Transitioning your HIV+ youth to healthy adulthood: a guide for health care providers. New York City: Children's Hope Foundation; 2007.

[21] Miles K, Edwards S, Clapson M Transition from paediatric to adult services: experiencesof HIV-positive adolescents. AIDS Care. 2004;16(3):305–14. 15203424 10.1080/09540120410001665312

[22] Cervia J Easing the transition of HIV-infected adolescents to adult care. AIDS Patient Care STDS. 2013;27(12):692–6. 24073595 10.1089/apc.2013.0253 PMC3868277

[23] Becker DF, Daley M, Green MR, Hendren RL Adolescent psychiatry. New York: The Analytic Press; 2003.

[24] Lee S. Achieving HR 90-90-90 in paediatric HIV: adolescence as the touchstone for transition success. J Int AIDS Soc. 2015;18(Suppl 6):20257. 26639113 10.7448/IAS.18.7.20257 PMC4670843

[25] Hosek SG, Zimet GD Behavioral considerations for engaging youth in HIV clinical research. J Acquir Immune Defic Syndr. 2010;54(Suppl 1):S25–30. 20571420 10.1097/QAI.0b013e3181e15c22

[26] Fair C, Allen H, Trexler C “I always wanted a big family because I lost mine”: A qualitative analysis of parenting perspectives among young parents with perinatally-acquired HIV. Abstract presented at AIDS 2016; 2016 Jul 18–22; Durban.

[27] Rydström LL, Wiklander M, Navér L, Ygge BM, Eriksson LE HIV-related stigma and health-related quality of life among children living with HIV in Sweden. AIDS Care. 2016;28(5):665–71. 26679064 10.1080/09540121.2015.1120267

[28] Dowshen N, Binns HJ, Garofalo R Experiences of HIV-related stigma among young men who have sex with men. AIDS Patient Care STDS. 2009;23(5):371–6. 19320600 10.1089/apc.2008.0256 PMC2856436

[29] Chiriboga CA, Fleishman S, Champion S, Gaye-Robinson L, Abrams EJ Incidence and prevalence of HIV encephalopathy in children with HIV infection receiving highly active anti-retroviral therapy (HAART). J Pediatr. 2005;146(3):402–7. 15756229 10.1016/j.jpeds.2004.10.021

[30] Paramesparan Y, Garvey LJ, Ashby J, Foster CJ, Fidler S, Winston A High rates of asymptomatic neurocognitive impairment in vertically acquired HIV-1-infected adolescents surviving to adulthood. J Acquir Immune Defic Syndr. 2010;55(1):134–6. 20733406 10.1097/QAI.0b013e3181d90e8c

[31] Laughton B, Cornell M, Boivin M, Van Rie A Neurodevelopment in perinatally HIV-infected children: a concern for adolescence. J Int AIDS Soc. 2013;16:18603. 23782482 10.7448/IAS.16.1.18603 PMC3687073

[32] Wood SM, Shah SS, Steenhoff AP, Rutstein RM The impact of AIDS diagnoses on long-term neurocognitive and psychiatric outcomes of surviving adolescents with perinatally acquired HIV. AIDS. 2009;23:1859–65. 19584705 10.1097/QAD.0b013e32832d924f

[33] Nichols SL, Bethel J, Garvie PA, Patton DE, Thornton S. Kapogiannis BG, et al Neurocognitive functioning in antiretroviral therapy-naïve youth with behaviorally acquired human immunodeficiency virus. J Adolesc Health. 2013;53(6):763–71. 23972941 10.1016/j.jadohealth.2013.07.006 PMC3878875

[34] Agwu A Thinking about the future: transition for Adolescents with HIV. Paper presented at: Conference on Retroviruses and Opportunistic Infections; 2016 2 22-26; Boston, MA.

[35] Remien RH, Mellins CA Long-term psychosocial challenges for people living with HIV: let’s not forget the individual in our global response to the pandemic. Aids. 2007;21(Suppl 5):S55–63. 10.1097/01.aids.0000298104.02356.b3 18090270

[36] Mellins CA, Malee KM Understanding the mental health of youth living with perinatal HIV infection: lessons learned and current challenges. J Int AIDS Soc. 2013;16:18593. 23782478 10.7448/IAS.16.1.18593 PMC3687078

[37] Bucek A, Cheng-Schien L, Benson S, Warne P, Abrams E, Elkington E, et al Antiretroviral treatment adherence, viremia, and psychiatric diagnosis throughout adolescence among perinatally HIV-infected youth. Abstract presented at AIDS 2016; 2016 Jul 18–22; Durban.

[38] Mellins CA, Elkington KS, Leu CS, Santamaria EK, Dolezal C, Wiznia A, et al Prevalence and change in psychiatric disorders among perinatally HIV-infected and HIV-exposed youth. AIDS Care. 2012;24(8):953–62. 22519762 10.1080/09540121.2012.668174 PMC3416047

[39] Mutumba M, Bauermeister JA, Elkington KS, Bucek A, Dolezal C, Leu CS, et al A prospective longitudinal study of mental health symptoms among perinatally HIV-Infected and HIV-exposed but uninfected urban youths. J Adolesc Health. 2016;58(4):460–6. 26873611 10.1016/j.jadohealth.2015.12.014 PMC4808472

[40] Ryscavage P, Anderson EJ, Sutton SH, Reddy S, Taiwo B Clinical outcomes of adolescents and young adults in adult HIV care. J Acquir Immune Defic Syndr. 2011 10 1;58(2):193–7. 21826014 10.1097/QAI.0b013e31822d7564

[41] Ryscavage P, Macharia T, Raphael L, Lovelace S, Tepper V, Redfield R Patterns of systemic hypertension among adults with perinatally-acquired HIV. Abstract presented at 23^rd^ Conference on Retroviruses and Opportunistic Infections; 2016 2 22-25; Boston, MA.

[42] Ryscavage P, Macharia T, Patel D, Palmeiro R, Tepper V Linkage to and retention in care following healthcare transition from pediatric to adult HIV care. AIDS Care. 2016;28(5):561–5. 26766017 10.1080/09540121.2015.1131967

[43] Gilliam PP, Ellen JM, Leonard L, Kinsman S, Jevitt CM, Straub DM Transition of adolescents with HIV to adult care: characteristics and current practices of the adolescent trials network for HIV/AIDS interventions. J Assoc Nurses AIDS Care. 2011 Jul-Aug;22(4):283–94. 20541443 10.1016/j.jana.2010.04.003 PMC3315706

[44] Murphy DA, Wilson CM, Durako SJ, Muenz LR, Belzer M Adolescent Medicine HIV/AIDS Research Network. Antiretroviral medication adherence among the REACH HIV-infected adolescent cohort in the USA. AIDS Care. 2001;13(1):27–40. 11177463 10.1080/09540120020018161

[45] Kahana SY, Jenkins RA, Bruce D, Fernandez MI, Hightow-Weidman LB, Bauermeister JA Structural determinants of antiretroviral therapy use, HIV care attendance, and viral suppression among adolescents and young adults living with HIV. Plos One. 2016;11(4):e0151106. 27035905 10.1371/journal.pone.0151106 PMC4817971

[46] Thompson MA, Mugavero MJ, Amico KR, Cargill VA, Chang LW, Gross R, et al Guidelines for improving entry into and retention in care and antiretroviral adherence for persons with HIV: evidence-based recommendations from an International Association of Physicians in AIDS Care panel. Ann Intern Med. 2012;5;156(11):817–33. 10.7326/0003-4819-156-11-201206050-00419 PMC4044043 22393036

[47] Fortenberry JD, Martinez J, Rudy BJ, Monte D Linkage to care for HIV-positive adolescents: a multisite study of the adolescent medicine trials units of the adolescent trials network. J Adolesc Health. 2012;51(6):551–6. 23174464 10.1016/j.jadohealth.2012.03.012 PMC3505853

[48] Sagrestano LM, Clay J, Finerman R, Gooch J, Rapino M Transportation vulnerability as a barrier to service utilization for HIV-positive individuals. AIDS Care. 2014;26(3):314–9. 23876086 10.1080/09540121.2013.819403

[49] Kreider AR, French B, Aysola J, Saloner B, Noonan KG, Rubin DM Quality of health insurance coverage and access to care for children in low-income families. JAMA Pediatr. 2016;170(1):43–51. 26569497 10.1001/jamapediatrics.2015.3028 PMC8011294

[50] Collaborative Initiative for Paediatric HIV Education and Research (CIPHER) Adolescent HIV Transition Workshop. 2015 3 28 [cited 2016 July 11]; Available from: https://www.iasociety.org/Web/WebContent/File/CIPHER_Adolescent_Transition_Meeting_Report_2015.pdf

[51] Valenzuela JM, Buchanan CL, Radcliffe J, Ambrose C, Hawkins LA, Tanney M, et al Transition to adult services among behaviorally infected adolescents with HIV–a qualitative study. J Pediatr Psychol. 2011;36(2):134–40. 19542198 10.1093/jpepsy/jsp051

[52] Vijayan T, Al B, Wagner K, Romano S, Wa A We never thought this would happen: transitioning care of adolescents with perinatally acquired HIV infection from pediatrics to internal medicine. AIDS Care. 2009;21(10):1222–9. 20024697 10.1080/09540120902730054 PMC2797130

[53] Sharma N, Willen E, Garcia A, Sharma TS Attitudes toward transitioning in youth with perinatally acquired HIV and their family caregivers. J Assoc Nurses AIDS Care. 2014;25(2):168–75. 23809660 10.1016/j.jana.2013.01.007

[54] Agwu AL, Lee L, Fleishman JA, Voss C, Yehia BR, Althoff KN, et al Aging and loss to follow-up among youth living with human immunodeficiency virus in the HIV research network. J Adolesc Health. 2015;56(3):345–51. 25703322 10.1016/j.jadohealth.2014.11.009 PMC4378241

[55] Fair CD, Sullivan K, Gatto A Indicators of transition success for youth living with HIV: perspectives of pediatric and adult infectious disease care providers. AIDS Care. 2011;23(8):965–70. 21390882 10.1080/09540121.2010.542449

[56] Campbell T The HIV journey from paediatric to adult services. [cited, 2016 8 22]; Available from: http://www.ukcab.net/wp-content/uploads/2013/07/The-HIV-journey-from-paediatric-to-adult-services.pdf

[57] Hussen SA, Chahroudi A, Boylan A, Camacho-Gonzalez AF, Hackett S, Chakraborty R Transition of youth living with HIV from pediatric to adult-oriented healthcare: a review of the literature. Future Virol. 2015;9(10):921–9. 25983853 10.2217/fvl.14.73 PMC4433446

[58] Bundock H, Fidler S, Clarke S, Holmes-Walker DJ, Farrell K, McDonald S, et al Crossing the divide: transition care services for young people with HIV-their views. AIDS Patient Care STDS. 2011;25(8):465–73. 21745141 10.1089/apc.2010.0279

[59] Kakkar F, Van Der Linden D, Valois S, Maurice F, Onnorouille M, Lapointe N, et al Health outcomes and the transition experience of HIV-infected adolescents after transfer to adult care in Québec, Canada. BMC Pediatr. 2016;16:109. 27457719 10.1186/s12887-016-0644-4 PMC4960665

[60] Fish R, Judd A, Jungmann E, O’leary C, Foster C Network HIVYP. Mortality in perinatally HIVinfected young people in England following transition to adult care: an HIV Young Persons Network (HYPNet) audit. HIV Med. 2014;15(4):239–44. 24112550 10.1111/hiv.12091

[61] Hope RL, Judd A, Foster C, Prime K, Jungmann E, Tookey P, et al Clinical outcomes among adults with perinatally-acquired HIV after transfer from pediatric care. Abstract presented at 23rd Conference on Retroviruses and Opportunistic Infections; 2016 2 22-25; Boston, MA.

[62] Righetti A, Prinapori R, Nulvesu L, Fornoni L, Viscoli C, Di Biagio A Transitioning HIV-infected children and adolescents into adult care: an Italian real-life experience. J Assoc Nurses AIDS Care. 2015;26(5):652–9. 26116060 10.1016/j.jana.2015.05.003

[63] Sainz T, Fernandez Mcphee C, Jimenez De Ory S, Gonzalez-Tome M, Rubio R, Bernardino J, et al Transition to adult units: situation and evolution of vertically HIV infected youths in Spain. Conference on Retroviruses and Opportunistic Infections (CROI), 2015 2 23-26, Seattle Abstract 918.

[64] Sainz T, Navarro ML HIV-Infected Youths: transition in Spain Compared to the Netherlands. Clin Infect Dis. 2017 1 15;64(2):230. 10.1093/cid/ciw702 27986681

[65] Fair CD, Sullivan K, Gatto A Best practices in transitioning youth with HIV: perspectives of pediatric and adult infectious disease care providers Psychology Health and Medicine. Psychology Health & Medicine. 2010;15(5):515–27. 10.1080/13548506.2010.493944 20835962

[66] Suris J-C, Akre C Key elements for, and indicators of, a successful transition: An international Delphi study. Journal of Adolescent Health. 2015;56:612–8. 26003575 10.1016/j.jadohealth.2015.02.007

[67] Lee L, Yehia BR, Gaur AH, Rutstein R, Gebo K, Keruly JC, et al The impact of youth-friendly structures of care on retention among HIV-infected youth. AIDS Patient Care STDS. 2016;30(4):170–7. 26983056 10.1089/apc.2015.0263 PMC4827281

[68] Judd A, Sohn AH, Collins IJ Interventions to improve treatment, retention and survival outcomes for adolescents with perinatal HIV-1 transitioning to adult care: moving on up. Curr Opin HIV AIDS. 2016 9;11(5):477–86. 27272537 10.1097/COH.0000000000000302

[69] Scharff DP, Mathews KJ, Jackson P, Hoffsuemmer J, Martin E, Edwards D More than Tuskegee: understanding mistrust about research participation. J Health Care Poor Underserved. 2010;21(3):879–97. 20693733 10.1353/hpu.0.0323 PMC4354806

[70] Writing group for the Kids to Adults Working Group and Data Management and Harmonisation Group in EuroCoord Children and young people with perinatal HIV in Europe: epidemiological situation in 2014 and implications for the future. Eurosurveillance. 2016;21(10):1–8. 10.2807/1560-7917.ES.2016.21.10.30162 26988197

[71] De Jose MI, Jiménez De Ory S, Espiau M, Fortuny C, Navarro ML, Soler-Palacín P, et al A new tool for the paediatric HIV research: general data from the cohort of the Spanish Paediatric HIV Network (CoRISpe). BMC Infect Dis. 2013;13:2. 23282073 10.1186/1471-2334-13-2 PMC3544738

[72] The Swiss HIV Cohort Study [cited 2016 8 31] Available from: http://www.shcs.ch/

[73] Transitioning HIV+ Adolescents to Adult Care: Exploring Adolescent and Adult Medicine Clinics Role in the Process [cited 2016 8 31]; Available from: https://clinicaltrials.gov/ct2/show/NCT02785302

[74] Tassiopoulos K, Patel K, Alperen J, Kacanek D, Ellis A, Berman C, et al Following young people with perinatal HIV infection from adolescence into adulthood: the protocol for PHACS AMP Up, a prospective cohort study. BMJ Open. 2016;6(6):e011396. 10.1136/bmjopen-2016-011396 PMC4908871 27288383

[75] Transitioning HIV-infected adolescents into adult care. 2011 [cited 2016 821]; Available from: http://www.hivguidelines.org/clinical-guidelines/adolescents/transitioning-hiv-infected-adolescents-into-adult-care

